# A star in the daylight clinical vignette: Lesional focal epilepsy with cerebral accessory falx

**DOI:** 10.1002/epd2.70235

**Published:** 2026-04-02

**Authors:** Kyle McGrath, Joel Kaye, John Paul Sheehy, Heather Ravvin McKee

**Affiliations:** ^1^ Department of Neurosurgery University of Cincinnati College of Medicine Cincinnati Ohio USA; ^2^ Department of Neurology, Epilepsy Division University of Cincinnati College of Medicine Cincinnati Ohio USA

**Keywords:** accessory falx, clinical vignette, epilepsy surgery, lesional focal epilepsy, refractory epilepsy

A 44‐year‐old right‐handed female with refractory focal epilepsy was found to have an exceedingly rare anatomic finding: an accessory falx (AF) cerebri. The AF was the abnormality corresponding to the area of seizure onset, and its resection resulted in seizure freedom. This rare congenital abnormality may represent a nidus for cortical irritability leading to focal epilepsy amenable to surgical resection and may be easily missed on magnetic resonance imaging (MRI) interpretation.

Her first seizure occurred during pregnancy in 2013. Seizure semiology included two types: focal seizures with preserved consciousness, characterized by a whole body “ripple of electricity” sensation descending from head to toe, accompanied by dizziness and nausea and focal seizures with impaired consciousness, beginning with the same aura and followed by inhalation, oral automatisms, and unresponsiveness. Both seizure types occurred weekly to monthly. Her seizures were initially controlled with levetiracetam monotherapy, but eventually became refractory to multiple agents, prompting neurosurgical referral.

Presurgical evaluation with scalp video‐EEG monitoring captured three focal seizures originating from the right posterior temporal region with right temporal lobe spread. Interictal findings were concordant with right temporal lateralized rhythmic delta activity, lateralized periodic discharges, isolated epileptiform discharges, and focal slowing shifting between posterior‐mid temporal maxima and anterior‐mid temporal maxima. Initial MRI findings showed possible right mesial temporal sclerosis, right lateral ventricle gray matter heterotopia, and atypical positioning of the inferior left frontal lobe into the middle cranial fossa. Positron emission tomography revealed subtly decreased uptake in the right lateral and mesial temporal regions (Figure 1A). Interictal magnetoencephalogram (MEG) confirmed right posterior temporal cortical irritability with a tight cluster of MEG dipoles (Figure 1B).

**FIGURE 1:**
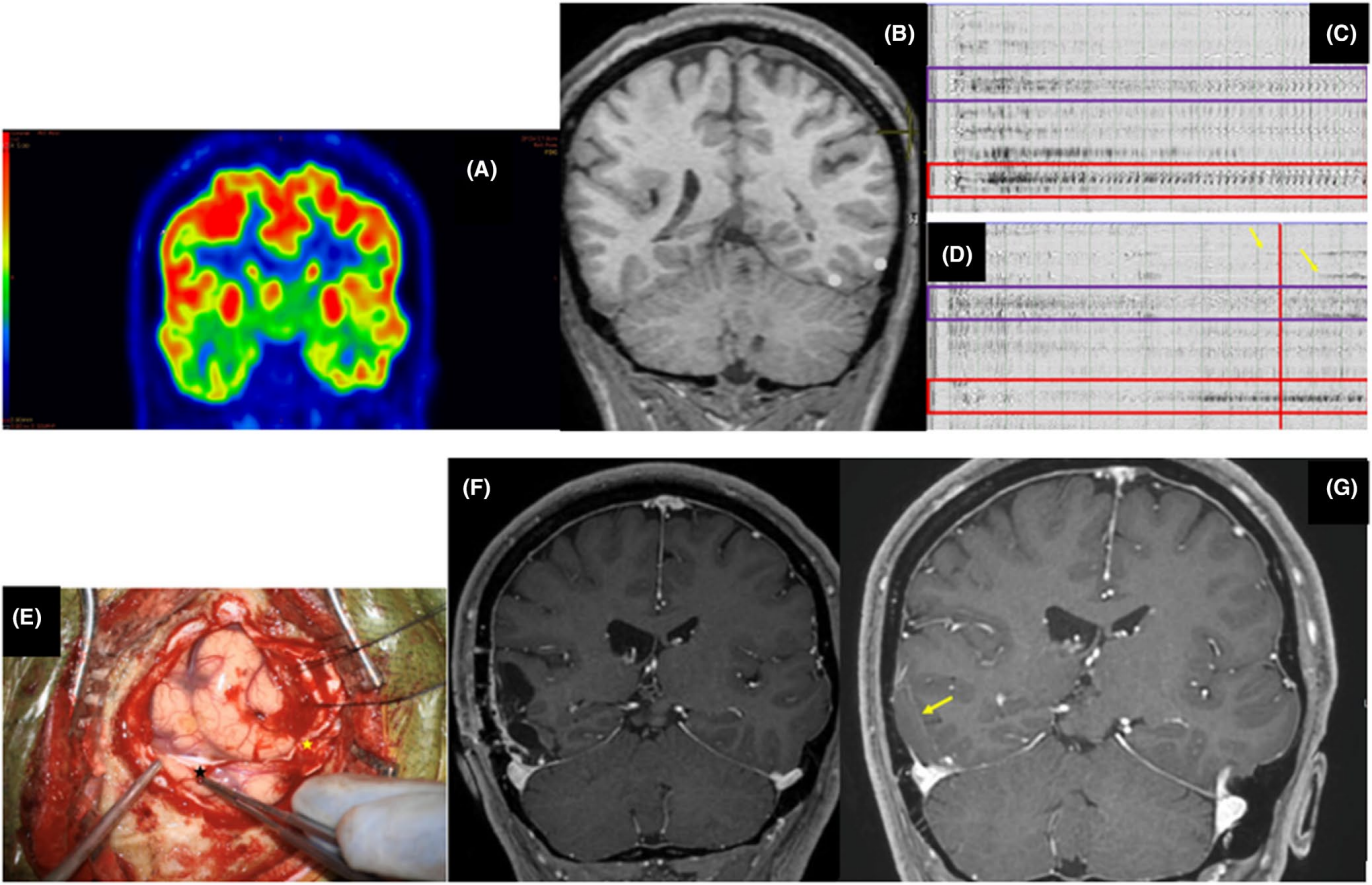
(A) Positron emission tomography revealing subtly decreased uptake in the right lateral and mesial temporal regions (yellow arrows). (B) Preoperative magnetoencephalogram (MEG)‐magnetic resonance imaging (MRI) indicates the locations of the tight cluster of MEG dipoles (white dots), closely corresponding to the location of the accessory falx. (C) Purple and red rectangles indicate seizure onset at depth electrodes in the posterior temporal region abutting the falx. Electrographic seizure on intracranial EEG without mesial temporal spread, seizure onset at right posterior MEG (R PMEG 1–10)‐purple rectangle and Right Middle Superior Temporal (R STM 1–8)‐red rectangle. (D) Clinical seizure on intracranial EEG with same areas of onset (clinical onset with arousal noted with vertical red line). Yellow arrows indicating spread to deepest hippocampal depth electrodes labeled Right Hippocampus and right posterior Hippocampus. Further spread continues (not included in diagram). See supplementary material for detailed SEEG information. (E) Intraoperative photograph demonstrates the accessory falx (AF) (black star indicating AF and yellow star indicating dural flap). (F) Postoperative MRI brain demonstrates absence of the AF and resection of the surrounding brain parenchyma within the seizure focus. (G) Preoperative coronal MRI demonstrates the AF (yellow arrow), seen retrospectively.

In May 2023, right hemispheric intracranial EEG monitoring (SEEG) recorded eight seizures, all with maximal, consistent, and evolving discharge from the posterior temporal region with or without rapid spread to mesial temporal structures (Figure 1C,D, Figure S1). Two were electrographic, and six had typical clinical semiology. Following this, a two‐stage surgical approach was planned to include both seizure onset and spread.

She underwent right mesial temporal laser ablation in November 2023. Three months later, she underwent right posterior temporal lobe resection, during which the AF was discovered intraoperatively overlying the epileptogenic parenchyma (Figure 1E). The AF overlying the cortex was divided (Figure 1F). Pathology of the resected parenchyma revealed fragments of cortical gray and white matter with moderate reactive gliosis without cortical dysplasia. Notably, the periventricular gray matter heterotopia was not the epileptogenic focus. Retrospective review of the preoperative MRI identified the AF (Figure 1G). She had no deficits following surgery (12/2023) with seizure freedom, Engel Class 1A.

The meningeal folds partition the intracranial contents. Variations of these folds have been reported. Klintworth demonstrated that the tentorium itself is not universally present across vertebrates, being absent in amphibians, fish, and reptiles, but well developed in birds and mammals.^1^ In some species, such as rats and mice, the tentorium remains unfused, whereas in humans and other mammals, it forms a midline crescentic partition posterior to the brainstem.^1^, ^2^ Variations of the falx have been more frequently documented, including its absence, duplication, and even triplication, often associated with vascular anomalies involving the occipital sinus^3^, ^4^ or meningeal arteries.^5^, ^6^ These variations can be associated with additional anomalies such as arachnoid cysts.^7^ Conversely, the falx cerebelli may be absent altogether in conditions such as Chiari malformation type II, where restricted posterior fossa growth can inhibit dural fold formation.^8^

While these reports emphasize the anatomic variability of both the tentorium and falx, an AF is rare, with only one reported case.^9^ The present case contributes to this body of knowledge, being the first report of an AF positioned over an epileptogenic focus. Resection of parenchyma in the region of this anomaly resulted in long‐term seizure freedom for our patient. Whereas prior literature has described falcine duplication primarily in relation to venous sinus anomalies or developmental malformations, our findings highlight an entirely new clinical dimension—that abnormal meningeal folds can be associated with epileptogenic cerebral cortex. This underscores the potential importance of recognition of dural anomalies, both on preoperative imaging and intraoperatively, as their significance may extend beyond anatomic variation to functional pathology.

## AUTHOR CONTRIBUTIONS

All authors listed contributed vital input to the finalized manuscript including concept, data collection, manuscript drafting, and providing essential edits.

## FUNDING INFORMATION

No funding was required for this case report.

## CONFLICT OF INTEREST STATEMENT

None.

## PATIENT CONSENT

Patient provided written consent to participate in the above case report.

## DISCLOSURE

The authors have no personal, financial, or institutional interest in any of the drugs, materials, or devices described in this article.

**Test yourself**

1. 44‐year‐old woman with refractory focal epilepsy undergoes surgical evaluation. Which of the following was ultimately identified intraoperatively as the structure overlying the epileptogenic cortex?
  - Periventricular nodular heterotopia
  - Accessory falx cerebri
  - Duplicated tentorium cerebelli
  - Arachnoid cyst
2. Which of the following best describes the seizure onset zone identified during intracranial EEG (SEEG) monitoring in this patient?
  - Left frontal lobe with contralateral spread
  - Right mesial temporal lobe exclusively
  - Right posterior temporal region with possible mesial temporal spread
  - Bilateral temporal lobes simultaneously
3. Which of the following statements regarding accessory falx cerebri (AF) is most accurate based on this case?
  - It is a common congenital variant frequently associated with epilepsy
  - It has been widely reported as a cause of mesial temporal sclerosis
  - It is an extremely rare anomaly that may serve as a nidus for epileptogenic activity
  - It is typically associated with Chiari II malformations

*Answers may be found in the* *Supporting Information*

## Supporting information


**Figure S1.** Two of her seizures on SEEG. Reference and ground electrodes are located in an easily accessible area on the scalp. Two screens shown for each seizure (A1/A2/B1/B2) to display all electrodes. Although other areas show interictal activity at or within the seizures, only the areas abutting the AF—R PMEG (purple) and R STM (red)—had consistency and evolution when evaluating all seizures. Electrode placement in order from top of EEG screen to the bottom: Right Amygdala (R Amy 1–16)—yellow bar, Right Hippocampus (R Hip 1–12)—yellow bar and yellow arrow, Right Posterior Hippocampus (R PHip 1–12)—yellow bar and yellow arrow, Right Posterior MEG (R PMEG 1–10)—purple rectangle (seizure onset and spread), Right Anterior Heterotopic Gray Matter (R AHGM 1–14)—blue bar, Right Middle Heterotopic Gray Matter (R MHGM 1–12)—blue bar, Right Posterior Heterotopic Gray Matter (R PGHM 1–12)—blue bar, Right Anterior Superior Temporal (R AST 1–6), Right Middle Superior Temporal (R STM 1–8)—red rectangle (seizure onset and spread), Right Posterior Superior Temporal (R PST 1–6), Right Temporal Occipital (R TO 1–12), (A) Electrographic, ictal speech, normal reading when tested at 8 s after seizure end. (B) Semiology: nocturnal, arousal (clinical onset shown with vertical red line) from sleep, oral automatism, later reports ripple of electricity occurred and “seizure washed through her body”, ictal speech—read during the discharge.

Data S2.

Data S1.

## Data Availability

Data sharing not applicable to this article as no datasets were generated or analysed during the current study.
