# Supplementary material for: A star in the daylight clinical vignette: Lesional focal epilepsy with cerebral accessory falx
Source: Epileptic Disord. 2026 Apr 2;28(4):1305–8. doi: 10.1002/epd2.70235 (PMC13499276; doi:10.1002/epd2.70235)
Supplement: Supplementary file 1 [file EPD2-28-1305-s002.pdf]

A Star in the Daylight Clinical Vignette: Lesional Focal Epilepsy with Cerebral Accessory Falx

Kyle McGrath, DO[1], Joel Kaye, MD[1], John Paul Sheehy, MD[1], Heather Ravvin McKee, MD, FAES[2]
[1]Department of Neurosurgery, University of Cincinnati College of Medicine, Cincinnati, Ohio, USA
[2]Department of Neurology, Epilepsy Division, University of Cincinnati College of Medicine, Cincinnati, Ohio, USA

Epileptic Disorders

ILAE

Introduction

- 44-year-old right-handed female with refractory focal epilepsy and abnormal MRI brain had an exceedingly rare finding, an accessory cerebral falx.

- The accessory falx was the primary abnormality corresponding to the area of seizure onset, and the resection of this area resulted in seizure freedom.

- Our findings suggest that this rare congenital abnormality, an accessory falx, may represent a nidus for cortical irritability leading to focal medication-resistant epilepsy amenable to surgical resection, and can be missed on MRI interpretation.

Case Report: Presurgical

- Her seizures became refractory to multiple antiseizure medications, prompting referral for epilepsy surgery evaluation.

- Presurgical evaluation revealed lesional epilepsy with initial MRI findings of possible right mesial temporal sclerosis (smaller volume of the right hippocampus), multiple foci of right lateral ventricle gray matter heterotopia, and a left frontal anatomical variant on MRI imaging. (Figure 1)

- There was no mention of an accessory falx on initial imaging interpretation.

Case Report: Surgical

- A two-stage surgical approach was planned to include both seizure onset and spread.
- She underwent right mesial temporal laser ablation in November 2023.
- A second surgery with right posterior temporal lobe resection was completed in February 2024 (Figure 1B), during which the accessory falx was discovered intraoperatively overlying the epileptogenic parenchyma (Figure 1C).
- Notably, the periventricular gray matter heterotopia was not the epileptogenic focus.
- Retrospective review of the preoperative MRI identified the anomalous falx-like structure (Figure 1D).
- She has no deficits following surgery with seizure freedom, Engel Class 1A surgical outcome.

Figure(s)

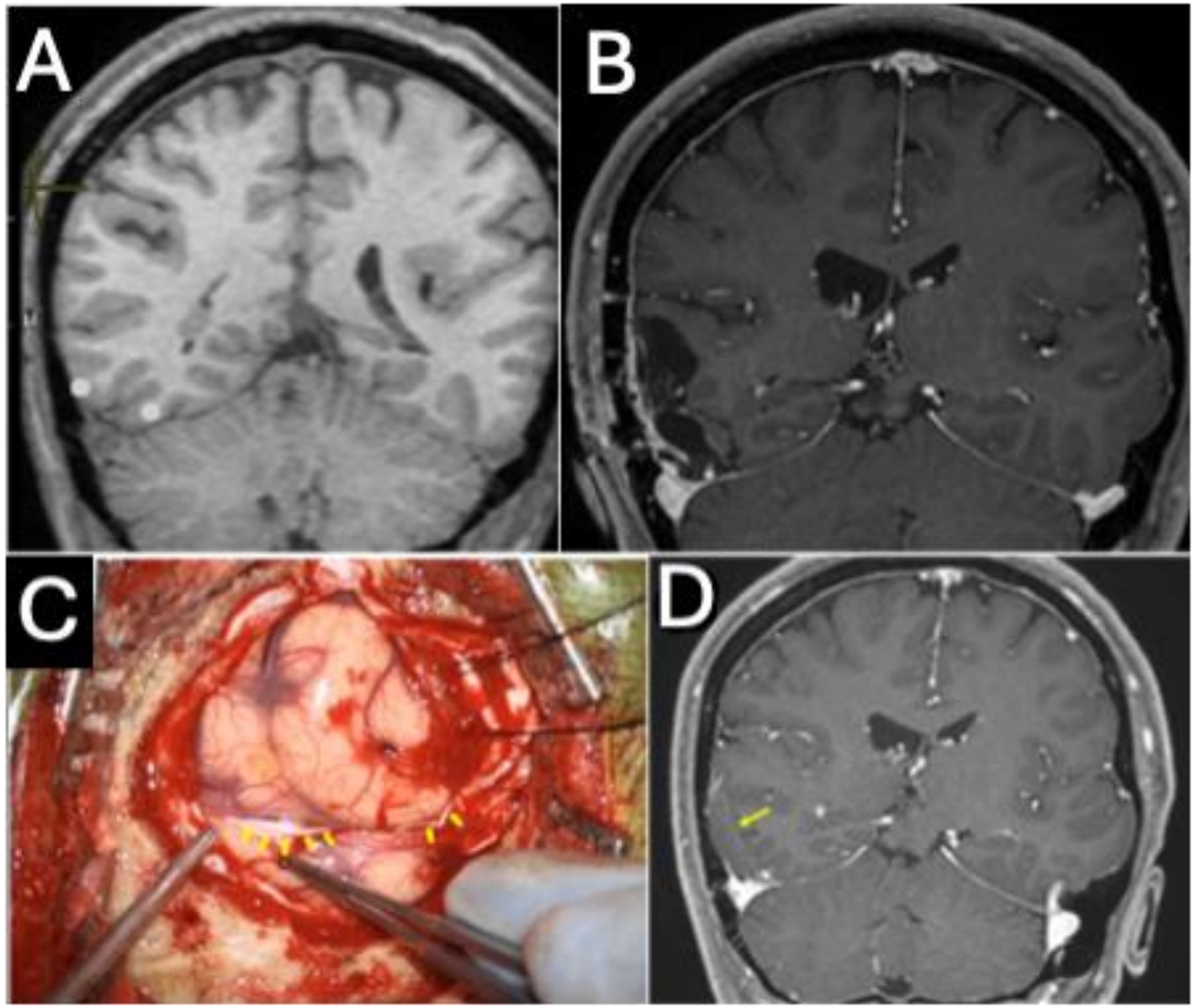

Conclusion

- Current reports emphasize that falx cerebelli variation is rare but documented.
- This is the first report of an accessory falx cerebri positioned over an epileptogenic focus and surgical resection of the adjacent tissue resulted in long term seizure freedom.
- Whereas prior literature has described falcine duplication primarily in relation to venous sinus anomalies or developmental malformations, our findings highlight an entirely new clinical dimension—that abnormal meningeal folds can be associated with epileptogenic cerebral cortex.
- This underscores the need for careful recognition of dural anomalies on imaging and intraoperatively, as their role may extend beyond anatomic variation to functional pathology.
