# Supplementary material for: A star in the daylight clinical vignette: Lesional focal epilepsy with cerebral accessory falx
Source: Epileptic Disord. 2026 Apr 2;28(4):1305–8. doi: 10.1002/epd2.70235 (PMC13499276; doi:10.1002/epd2.70235)
Supplement: Supplementary file 2 [file EPD2-28-1305-s001.docx]

**Supplementary material:**

Figure 2. Two of her seizures on SEEG. Reference and ground electrodes are located on easily accessible areas on the scalp. Two screens shown for each seizure (A1/A2/B1/B2) to display all electrodes. Although other areas show interictal activity at or within the seizures, only the areas abutting the AF—R PMEG (purple) and R STM (red)—had consistency and evolution when evaluating all seizures.

Electrode placement in order from top of EEG screen to the bottom:

Right Amygdala (R Amy 1-16)-yellow bar

Right Hippocampus (R Hip 1-12)-yellow bar and yellow arrow

Right Posterior Hippocampus (R PHip 1-12)-yellow bar and yellow arrow

Right Posterior MEG (R PMEG 1-10)-purple rectangle (seizure onset and spread)

Right Anterior Heterotopic Gray Matter (R AHGM 1-14)-blue bar

Right Middle Heterotopic Gray Matter (R MHGM 1-12) -blue bar

Right Posterior Heterotopic Gray Matter (R PGHM 1-12) -blue bar

Right Anterior Superior Temporal (R AST 1-6)

Right Middle Superior Temporal (R STM 1-8)-red rectangle (seizure onset and spread)

Right Posterior Superior Temporal (R PST 1-6)

Right Temporal Occipital (R TO 1-12)

2A) Electrographic, ictal speech, normal reading when tested at 8 seconds after seizure end

2B) Semiology: nocturnal, arousal (clinical onset shown with vertical red line) from sleep, oral automatism, later reports ripple of electricity occurred and “seizure washed through her body”, ictal speech—read during the discharge
