# Supplementary material for: A star in the daylight clinical vignette: Lesional focal epilepsy with cerebral accessory falx
Source: Epileptic Disord. 2026 Apr 2;28(4):1305–8. doi: 10.1002/epd2.70235 (PMC13499276; doi:10.1002/epd2.70235)
Supplement: Supplementary file 3 [file EPD2-28-1305-s003.docx]

**TEST YOURSELF**

**Answers**

**1. B.** Accessory falx cerebri

**2. C.** Right posterior temporal region with possible mesial temporal spread

**3. C.** It is an extremely rare anomaly that may serve as a nidus for epileptogenic activity
